# Two-stage Enteric Exclusion to Salvage a Pancreas Transplant After an Early Post-transplant Leak

**DOI:** 10.7759/cureus.5379

**Published:** 2019-08-13

**Authors:** George Rofaiel, Gilbert Pan, Eryberto Martinez, Robin Kim, Jeffrey Campsen

**Affiliations:** 1 Surgery, University of Utah School of Medicine/Huntsman Cancer Institute, Salt Lake City, USA

**Keywords:** pancreas transplant, transplantation, pancreas

## Abstract

Early technical complications after pancreas transplantation are almost always unsalvageable. The two most common complications are vascular thrombosis and duodenal anastomotic leaks. We present a case of a duodenal stump leak that led to a large abscess and severe sepsis. The pancreas was salvaged by repairing the leak and creating a proximal diverting ileostomy. Several months later, the ileostomy was reversed. This was done by creating a defunctionalized Roux limb to exclude the pancreas. The patient healed well and continued to enjoy excellent glucose control.

## Introduction

Whole pancreas transplantation has become an accepted form of treatment for type I diabetes mellitus. Based on Organ Procurement and Transplantation Network (OPTN) data as of May 22, 2019, 8,789 pancreas and 23,959 simultaneous kidney-pancreas transplants have been performed since January 1988. In 2018, there have been 192 pancreas and 836 kidney-pancreas transplants reported. Early graft loss is rare but is generally related to technical complications. Such complications are most commonly related to vascular thrombosis and intestinal anastomotic leaks. Traditionally, these complications are grave and graft salvage is not attempted. Retransplantation is also seldom attempted given the morbidity of the first operation.

We present a single case experience where a duodenal stump leak occurred early after pancreas transplantation. Transplant pancreatectomy was successfully avoided using a nuanced technique.

## Case presentation

**Figure 1:**
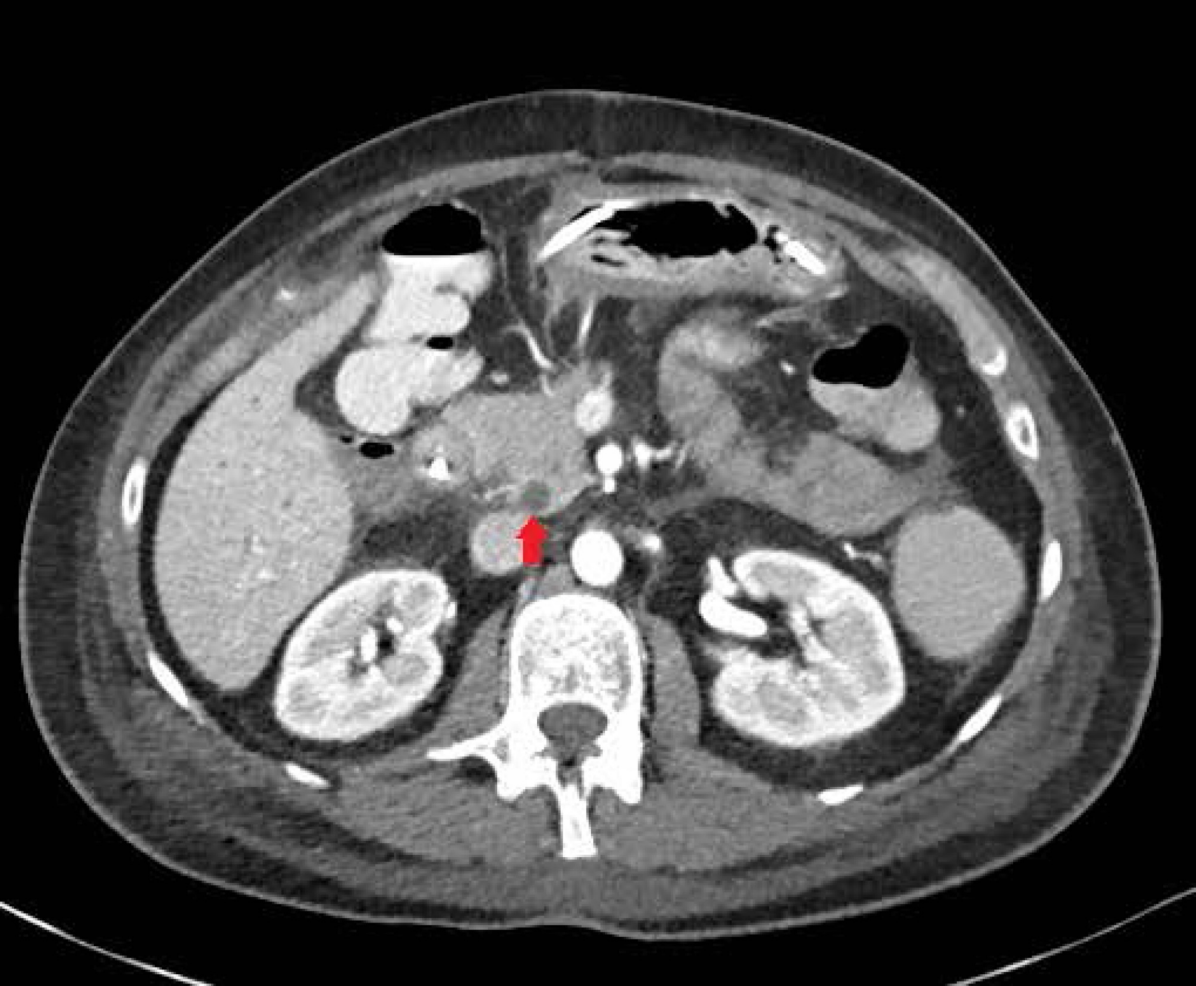
Abscess identified from CT imaging

**Figure 2:**
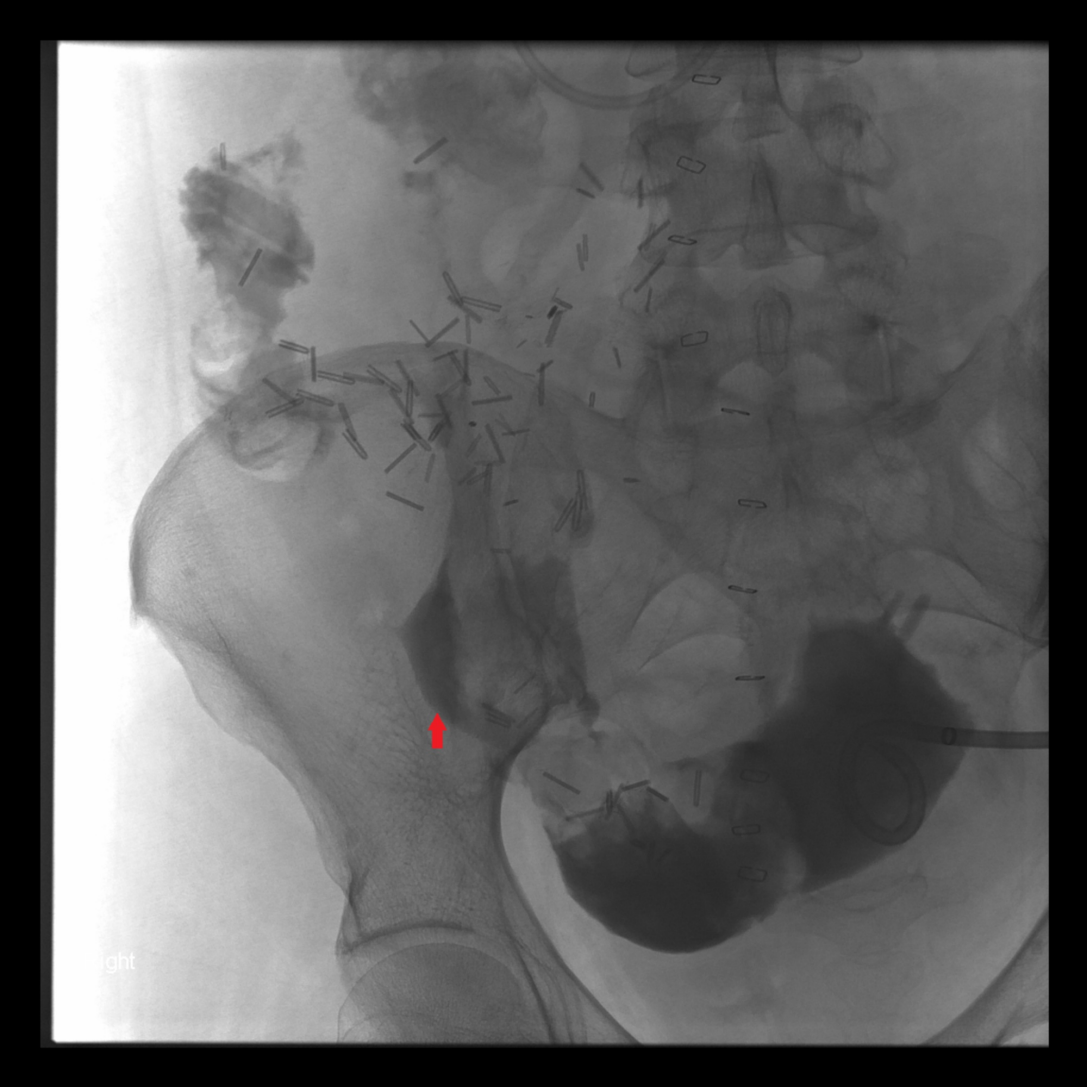
Contrast flowing into the intestines

The patient is a middle-aged female with type I diabetes mellitus. She suffered from difficulties with glucose control and hypoglycemic unawareness. She also developed the sequelae of diabetes, including retinopathy and peripheral neuropathy. As a result, the patient received a pancreas transplant. The patient had preserved kidney function with a glomerular filtration rate (GFR) of 107 ml/min and a protein to creatinine ratio of 138 mg/g. Vascular reconstruction was performed using a standard donor iliac Y-graft to the superior mesenteric and splenic arteries on the graft end. The common iliac artery of the donor was reconstructed to that of the recipient. The donor portal vein was anastomosed into the inferior vena cava. The duodenum of the donor was then drained using a standard two-layer, hand-sewn anastomosis to the recipient ileum. Initially, the patient had an uneventful postoperative course. About a month later, she presented with increasing pain and diarrhea. Cross-sectional imaging revealed an abscess (Figure 1). Upon drainage, the abscess was found to communicate with the intestinal tract, and a leak was diagnosed (Figure 2). An exploratory laparotomy was performed to assess the findings.

**Figure 3:**
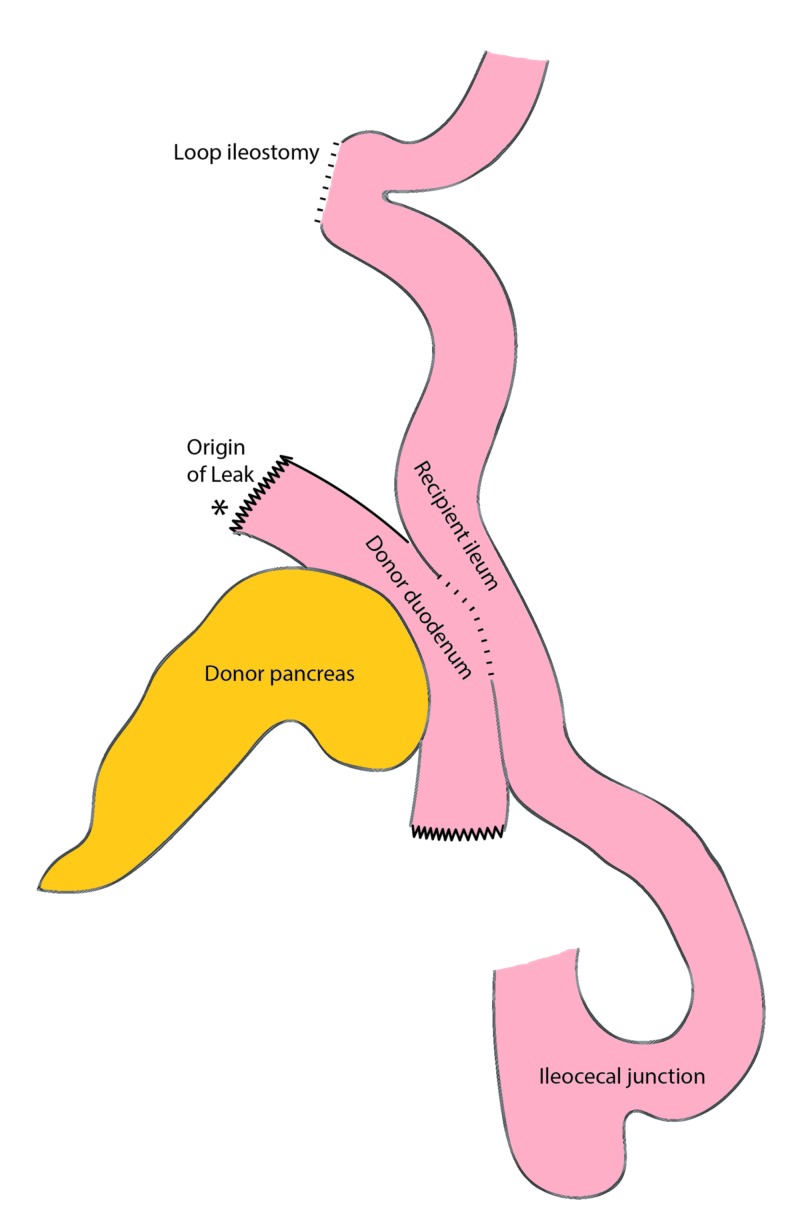
Original illustration of diverting loop ileostomy

**Figure 4:**
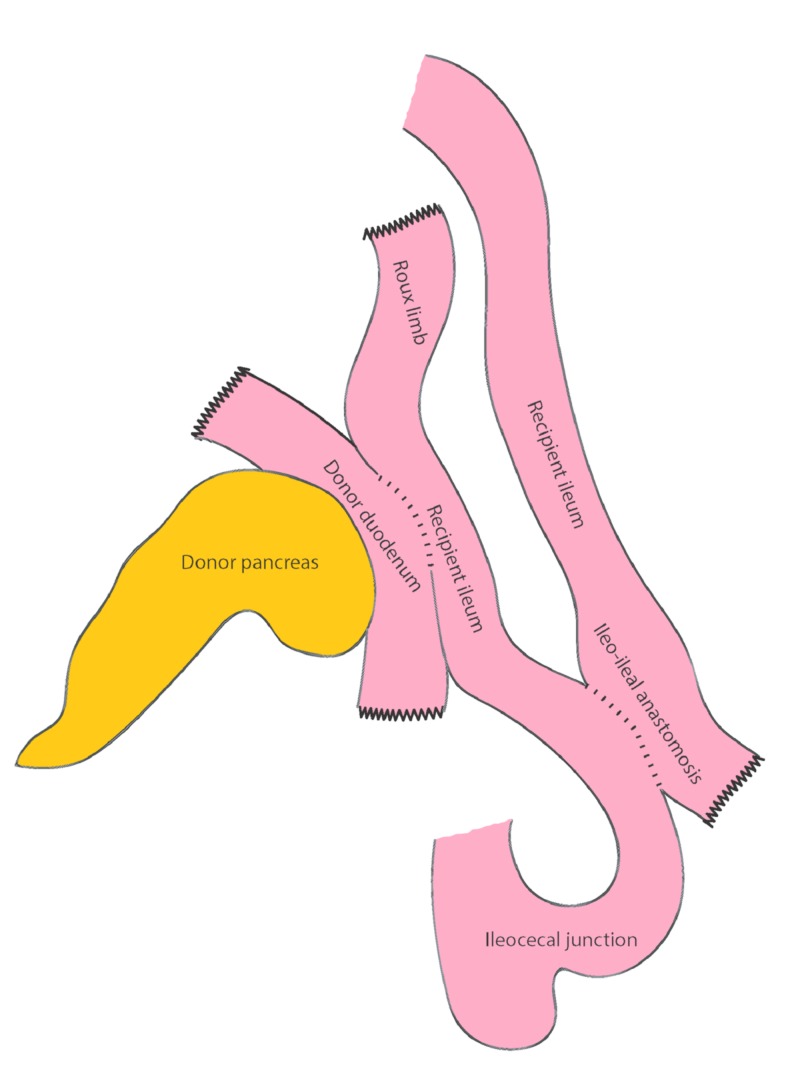
Original illustration of ileostomy takedown

She was found to have a large abscess cavity, and after adequate drainage, we did not find a leak at the duodenal anastomosis. The staple line at the proximal duodenum, however, had a 1-cm gap in it. The graft was initially thought to be unsalvageable. The edges of the leak were inflamed and devitalized. With intestinal contents passing by, any primary repair was guaranteed to fail. There was also not a precedent for a diversion this early after transplantation. We eventually decided to repair the defect with running non-absorbable monofilament stitches and performed a diverting loop ileostomy (Figure 3). The patient healed well, and the drains were removed. The pancreas continued to function well with excellent glucose control. Eight months later, we performed a planned revision of the ileostomy. We feared a simple closure of the ileostomy would reproduce the leak, so we decided to exclude the pancreas by taking down the ileostomy and performing an ileoileal anastomosis distal to the donor duodenum (Figure 4). This essentially produced a defunctionalized Roux limb to drain the pancreas. The patient healed well and was discharged a few days later on a regular diet. Several months later, she is reported to have an Hgb A1C of 5.3 % and a fasting C-peptide of 1.4 ng/ml. The patient continues to enjoy excellent glucose control from the pancreas and normal gastrointestinal function.

## Discussion

Pancreas transplantation has long been the most effective therapy for type I diabetes mellitus [1-3]. This is especially true for patients who develop hypoglycemic unawareness and other complications associated with end-organ damage [3]. In fact, some end-organ damage has been shown to reverse after successful pancreas transplantation, including nephropathy [4], retinopathy [5-6], and neuropathy [7]. Kidney function seems to be preserved for longer after simultaneous pancreas-kidney transplantation than pancreas transplantation alone [8]. Solitary pancreas transplantation in well-selected patients reverses diabetic complications, including proteinuria [9]. Our patient had a preserved GFR of 74 ml/min at a year post-transplant, with no change in proteinuria at 182 mg/g. She continues to maintain such function at the time of this publication close to two years out.

The surgical complications of pancreas transplantation have been well-described [10-11]. The most concerning complications are vascular thrombosis and duodenal leaks, historically leading to unsalvageable grafts in general [11]. Duodenal stump leaks have been reported to occur in 5% to 20% of all pancreas transplant recipients, including pancreas alone transplant, pancreas after kidney transplant, and simultaneous kidney-pancreas transplant [12]. Mauricio and Cesar described a case where a leak occurred after enteric conversion [13]. In this particular scenario, a well-healed pancreas was converted from bladder drainage to enteric drainage, and then an anastomotic leak occurred. The technique used was relatively similar to ours; however, the reported leak was minor and occurred neither immediately after transplantation nor after the induction of immunosuppression. Also, the graft had been well-incorporated.

The principle of enteric diversion for a leak is a well-established one. It has been long-utilized in perforated diverticulitis and perforated duodenal ulcers [14-15]. The principle of staged surgery in abdominal catastrophes has also been established [16-18]. The nuance here is applying these principles in salvaging a pancreas transplant. This was successful and arguably life-prolonging for the patient.

## Conclusions

Duodenal leaks are devastating complications after pancreas transplantation. Drainage, repair, and diversion can be utilized to salvage the transplant. These principles are applicable to other types of intestinal leaks, such as perforated acute diverticulitis, as well. This case report illustrates that two-staged diversion followed by revision can be safely and effectively used in salvaging leaks early after pancreas transplantation. Early recognition of the complication and swift salvage are paramount to success. Further case reports and series will be helpful in establishing the reliability and reproducibility of this technique.
